# Development and validity evidence on the scale of perceived social support for university students (EPSSEU) during the period of social restrictions

**DOI:** 10.1186/s12889-024-18882-3

**Published:** 2024-06-01

**Authors:** Thaís Calcagno Vidon Bruno, Isis Danyelle Dias Custódio, Luiz Antônio Alves de Menezes-Junior, Adriana Lúcia Meireles, Ana Cláudia Morito Neves, Sabrina Martins Barroso, Júlia Cristina Cardoso Carraro

**Affiliations:** 1 Graduate Program in Health and Nutrition, School of Nutrition, Federal University of Ouro Preto, Minas Gerais, Ouro Preto, Brazil; 2 Federal University of Uberlândia, Uberlândia, Minas Gerais Brazil; 3 Federal University of Triângulo Mineiro, Uberaba, Minas Gerais Brazil; 4 Department of Clinical and Social Nutrition, School of Nutrition, Federal University of Ouro Preto, Minas Gerais, Ouro Preto, Brazil; 5 Group for Research and Teaching in Nutrition and Collective Health, Federal University of Ouro Preto, Ouro Preto, Minas Gerais, Brazil; 6 Postgraduate in Statistics and Quantitative Modeling, Federal University of Santa Maria, Santa Maria, Rio Grande Do Sul, Brazil

**Keywords:** Students, Social support, Social restrictions

## Abstract

**Aim:**

This study aims to validate a Perceived Social Support Scale for University Students (EPSSEU) during periods of social restrictions, by focusing on family and university support.

**Subject and methods:**

This cross-sectional study was conducted with undergraduate students from a public higher education institution. The college students who participated in the study—1353 at baseline and 378 after 6 months—answered a virtual questionnaire containing questions on: sociodemographic and lifestyle data, items proposed for the EPSSEU, Satisfaction with Social Support Scale (ESSS), and Depression, Anxiety and Stress Scale (DASS-21). Exploratory factor analysis, Cronbach’s alpha reliability analysis, as well as discriminant, convergent, and known-group validations were performed.

**Results:**

The results showed two factors support from: i) the university and ii) friends and family— which explained 61.82% of the variance in the data. The EPSSEU showed good reliability (Cronbach’s alpha = 0.796) as well as validity, with higher scores among individuals without depression, anxiety, or stress.

**Conclusion:**

The EPSSEU shows adequate psychometric qualities and may be a useful instrument for assessing university students’ social support in pandemics, social distancing, and remote teaching contexts.

**Supplementary Information:**

The online version contains supplementary material available at 10.1186/s12889-024-18882-3.

## Introduction

In December 2019, Coronavirus Disease 2019 (COVID-19)—caused by a new virus—was declared a pandemic by the World Health Organization [1–3]. To prevent its spread, strict control measures—isolation and social distancing, closing of universities, prohibition of events with crowds, and restrictions on travel and public transportation—were implemented [4].

Previous infectious disease epidemics, such as the Severe Acute Respiratory Syndrome, Ebola, and Middle East Respiratory Syndrome, have shown detrimental effects—elevated stress levels and emerging psychological distress in the population [5–7]. Some previous studies have already shown that college students are at a higher risk of developing mental distress compared to the general population [8, 9], and the COVID-19 pandemic seems to have exacerbated this scenario. Further, the pandemic and restrictive measures adopted to control the disease have also been linked to the development of various psychological problems in college students [10]. A survey in the United States (US) found that 71% of the college students surveyed, revealed increased stress and anxiety owing to the pandemic, and 44% mentioned having depressive thoughts [11, 12].

Another study conducted by Wang et al. [13], also in the US, reported that 48% and 38% of college students had moderate to severe levels of depression and anxiety, respectively [13]. A study in Saudi Arabia that assessed the psychological conditions of college students, found that 26% and 22% of them had symptoms of anxiety and stress, respectively. In addition, students who reported that they did not receive emotional support from their family, university, and society had a higher risk of developing psychological problems [14].

A factor that may help to explain this increase in illnesses is the perceived lack of social support—defined as the assistance and protection provided to other people [15, 16]. The assistance could be real—financial help, or non-real—emotional help, where protection may present itself as shielding people from the adverse effects of stress [17, 18]. Such needs can be met through stress-reducing functions, providing practical help, advice, or information, assisting in problem solving or wishful thinking, or ameliorating the impacts of problematic events [19].

Social support acts as a mediator between stress arising from the demands of the environment and mental health. In the context of the COVID-19 pandemic, which has contributed to a chaotic and hectic living environment for many people, there has been an increased search for coping strategies and an increased need for support from family, friends, and teachers [20].

However, the perception of support is subjective, as it depends on needs and expectations in relation to the situation experienced [21], and is also dependent on the actors involved [22].

Although individuals generally seek support from their families and social networks subsequent to trauma, the severity of traumatic experiences can negatively affect their perceived social support, and directly reduce their support seeking behaviors [23, 24]. Amid such a depressing state of life, social support is considered an essential positive resource, that can redeem individuals from life’s adverse consequences [25].

However, assessing perceived social support during periods of social restriction can be challenging, as instruments typically use dimensions related to physical contact [26–28], which was dramatically reduced at the onset of the COVID-19 pandemic. Furthermore, it was most likely that the perceived social support of university students during the pandemic context, extended beyond the needs of family and friend relationships, to the university context, as remote classes and difficulties experienced in the study routine were causes of great stress and anxiety, in this population group [28–31].

In this context, some scales—Satisfaction with Social Support Scale (ESSS) [28], which assesses social support through four dimensions: satisfaction with friends, intimacy, satisfaction with family, and social activities—have already been validated to measure social support, but as previously reported, the ESSS does not allow assessment in times of social restrictions, as in the COVID-19 pandemic, and has also not been validated in Brazilian university students. Therefore, this study aims to validate the Scale of Perception of Social Support for University Students (EPSSEU) during periods of social restrictions.

## Methods

### Design and study population

This study forms part of a larger project entitled “Effect of the COVID-19 pandemic on the mental and nutritional health, and home food environment of the academic community: A longitudinal evaluation of the PADu-COVID project,” conducted between July 2020 and February 2021, with undergraduates of a public higher education institution in the southeastern region of Brazil. The research was approved by the Universidade Federal de Ouro Preto’s Research Ethics Committee under CAAE 19467919.5.0000.5150 and opinion 3.784.449. Participants had access to the online Informed Consent Form (ICF), containing explanations about the research objectives and the request for authorization to use the data, and only those who confirmed their consent had access to the research.

### Data collection

Data were collected at four time points: T0 (baseline), T1 (after 3 months), T2 (after 6 months), and T3 (after 9 months). However, for the present study, only T0 and T2 data were used, performing analyses from a cross-sectional study.

Data collection was performed in a virtual environment, using a self-administered, confidential questionnaire, sent by e-mail to all regularly enrolled undergraduate students, and made available through an online platform (Google Forms).

All undergraduate students of the higher education institution, who filled out the questionnaire within 4 weeks, after the invitation was sent, were included in the study, whereas those who did not respond adequately to questions related to the study’s main question were excluded. After the first invitation, three reminders—one per week, on alternate days—were sent to those, who had not answered the questionnaire. The survey was disseminated through all social networks linked to the educational institution.

### Instruments

The questionnaire sent at T0 contained the EPSSEU, as well as sociodemographic and economic data. At T2, the students who had responded to T0 were sent a new questionnaire, again containing the EPSSEU, in addition to the Depression, Anxiety, and Stress Scale (DASS-21) [32] and ESSS [28].

The sociodemographic and economic data collected were: age (continuous); sex (female or male); marital status (single/widowed/divorced, or married/stable union); household (alone/in a pension, hotel and others/with family, or a sorority/housing or apartment/house with other people); education of the head of the household (no education or incomplete primary education, elementary school, high school, and college education); and per capita income (less than, greater than, or equal to one minimum wage).

For analyzing the symptoms of depression, anxiety, and stress, the Portuguese version of the DASS-21 proposed by Vignola and Tucci [32] was used. It contains 21 questions and a 4-point response format, ranging from 0 (did not apply at all) to 3 (applied a lot or most of the time). Scores from the scale were categorized according to the presence or absence of symptoms of depression, anxiety, and stress, with cut-off points considered to be scores of 10, 8, and 15 or higher, respectively. In the present sample, the instrument’s Cronbach’s alpha was 0.95.

The ESSS, that was used for convergent validation, consists of 15 items divided into 4 subscales: satisfaction with friends, intimacy, satisfaction with family, and social activities. The answers were presented in a 5-point format, ranging from 1 (strongly agree) to 5 (strongly disagree), with the highest score corresponding to a perception of greater social support [28]. In the present sample, the Cronbach’s alpha was 0.86 for the total instrument, and 0.83, 0.79, 0.84, and 0.61 based on its factors of satisfaction with friendships, intimacy, satisfaction with family, and social activities, respectively.

### Statistical analysis

Descriptive analyses were carried out by calculating the absolute and relative frequencies, means (± standard deviations), or medians (p25 and p75). The Shapiro–Wilk test was used to verify the normality of continuous data. With the data obtained at T0, exploratory factor analysis was performed, using factor software to evaluate the EPSSEU’s factor structure. The analyses was implemented using a polychoric correlation matrix and the robust diagonally weighted least squares extraction method [33]. Sample adequacy was tested using the Kaiser–Meyer–Olkin (KMO) indicator, where values greater than 0.5 were considered acceptable, and Bartlett’s test of sphericity, where a *p* < 0.05 was considered acceptable [34, 35]. The decision on the number of factors to be retained was made using the parallel analysis technique, with random permutation of the observed data [36], and the Robust Promin rotation method [37]. Factor loading values above 0.30 were considered [35].

Confirmatory factor analysis was used to assess the model fit by means of the Root Mean Square Error of Approximation (RMSEA), Comparative Fit Index (CFI), and Tucker-Lewis Index (TLI). The ideal values are considered to be less than 0.08 [15] for RMSEA, and above 0.90, or preferably 0.95 for CFI and TLI. Factor stability was assessed using the H-index [37] and composite reliability [38]. The H-index assesses how well a set of items represents a common factor, with values greater than 0.80, suggesting a well-defined latent variable, that is likely to be stable across different studies [37].

Internal consistency was assessed using the Cronbach’s alpha coefficient (total, per domain, and partial, based on the possibility that each item is deleted), as well as McDonald’s Omega (ω). As the interpretability of the Cronbach’s alpha can be restricted in multidimensional scales, McDonald’s ω was also calculated for the total scale, as it is considered a reliability index, with a lower risk of over or underestimation of reliability in such cases [8, 39, 40]. For both measures, values equal to or greater than 0.70 were considered acceptable [41].

The data collected at T2 were used for the analyses of internal consistency, as well as convergent and discriminant validity, for which Spearman’s correlation was used. Discriminant validity was assessed by correlating the score of each scale item with the score of the domain to which it belongs or does not belong, and the total, whereas convergent validity was assessed through the correlation between the total scores or dimensions of the EPSSEU and ESSS. Given the number of statistical analyses performed, statistical significance was set at 1%, and the following interpretations of correlation strength were considered: *r* = 0.10 to 0.29 (low), 0.30 to 0.49 (moderate), > 0.50 (high) [21].

Validation by known groups was also performed using the Student’s t-test or Mann–Whitney test, according to data normality for comparison of means/averages between the overall scores and dimensions of the EPSSEU and ESSS scales, regarding the presence or absence of anxiety, stress, and depression. For data analysis, STATA software, version 13.0, was used, and a 5% significance level was considered for all analyses, except correlation analyses.

### Description of the population

Among the 1353 students of both genders evaluated at T0, 66.30% were women, 91.97% single, 51.81% had a per capita income equal to or higher than one salary, and 50.71% lived with other people (dorms, housing, apartments/houses). Their mean age was 24.07 ± 5.71 years, and as regards the level of education of the person responsible for the family: 10.94% had no education or incomplete primary schooling, 17.15% elementary school, 34.81% high school, and 37.10% college education.

Of the 378 students of both genders, who participated in T2, women comprised 67.46%, those who were single 66.76%, and those living with other people (sorority house, rooming house, apartment/house) 49.20%. Their mean age was 24.88 ± 6.48 years, and 26.98% had a per capita income equal to, or higher than one salary. As regards, the level of education of the person responsible for the family, it included 12.70% with no education or incomplete elementary school education, and 16.93%, 33.86%, and 36.51% with elementary school, high school, and college education, respectively. 

## Results

### Construction of the scale

#### Construct validation

This step was carried out to evaluate the structural components of the perception of social support based on the theoretical construct. It used questions from the ESSS [28] for assessing the reality of social restrictions, and also included questions regarding the university environment to evaluate the extent to which the elaborated items correlated with these components. Only the first question remained the same as the ESSS, while the others were changed. Responses were rated using a 5-point Likert format, with responses ranging from 1 (strongly agree) to 5 (strongly disagree), and items 2 through 9 were scored in reverse. The scale’s total score ranged from 9 to 45 points, with a higher score corresponding to a higher perception of social support.

#### Psychometric properties

**Fig. 1:**
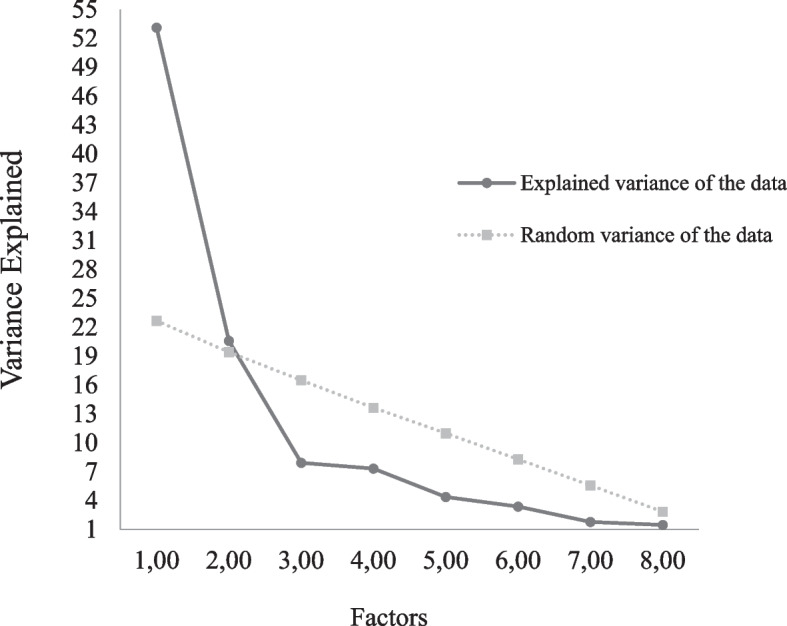
Results of the parallel analysis, according to the variance of the data of each factor of the scale of perceived social support for college students (EPSSEU)

Bartlett’s test of sphericity (5333.8, gl = 36, *p* < 0.001) and KMO (0.80) suggested a good interpretability of the correlation matrix of the items. Parallel analysis suggested the presence of two most representative factors for the data (Fig. 1): satisfaction with university support (US) and satisfaction with friends/family support (FFS).

The structure and factor loadings of the EPSSEU items are shown in Table 1, along with the composite reliability indices, replicability estimates of the factor scores, and variance explained by the eigenvalues (H-index).

**Table 1:** Factor structure of the scale of perceived social support for university students (EPSSEU) (*n* = 1353)

Items	Factorial loadings	
	US	FFS
Sometimes I feel alone in the world and without support	-0.109	**0.679**
I feel that I can count on concrete help from the people I live with during the pandemic	-0.064	**0.871**
I have several people to talk to, even by phone, messaging apps or social networking, in case I feel lonely	-0.037	**0.809**
I am satisfied with the way the people at home are dealing with the pandemic	0.024	**0.569**
My family members have helped me in whatever I need during the pandemic	0.006	**0.806**
My friends have helped me in whatever I need during the pandemic	0.025	**0.737**
I feel that my professors are looking for a way to maintain my learning and bond with the university during the pandemic	**0.879**	-0.024
I feel that the university is looking for a safe and efficient way to maintain my learning and bonding during the pandemic	**0.871**	-0.001
I have received the financial support I need during the pandemic	0.118	**0.409**
Composite Reliability	0.867	0.874
H-latent	0.867	0.902
H-observed	0.830	0.900
Explained variance (%)	61.82	

*FFS* Satisfaction with friends/family, *US* Satisfaction with university

The items showed adequate high factor loadings on their respective factors, with US being determined by items 7 and 8, and FFS by items 1, 2, 3, 4, 5, 6, and 9. The combined factors accounted for 61.82% of the explained variance in data.

No pattern of cross-loadings (items with factor loadings above 0.30 on more than one factor) was found. The composite reliability of the factors was also adequate (> 0.70) for all factors, and the replicability measure of the factor structure suggests that all factors may be replicable in future studies (H > 0.80).

Confirmatory factor analysis at T0 showed adequate fit indices, except for RMSEA (RMSEA = 0.110; CFI = 0.969; TLI = 0.941). At T2, the analysis also showed adequate fit indices, except for RMSEA (RMSEA = 0.107; CFI = 0.977; TLI = 0.956).

The reliability analysis indicated a McDonald’s ω value of 0.835. Table 2 presents the Cronbach’s alpha at the two collection times, and it can be seen that all coefficients showed good to excellent internal consistency. Through discriminant validity, it was observed that the items were correlated with the dimensions to which they belonged (Table 3).

**Table 2:** Reliability as internal consistency: total, domain and partial Cronbach’s alpha of the EPSSEU

	Cronbach’s alpha (T0)	Cronbach’s alpha (T0)
s/1	0.736	0.800
s/2	0.759	0.784
s/3	0.762	0.790
s/4	0.781	0.811
s/5	0.758	0.788
s/6	0.764	0.800
s/7	0.794	0.806
s/8	0.791	0.810
s/9	0.791	0.826
FFS	0.810	0.810
US	0.803	0.856
Total	0.796	0.819

*FFS* Satisfaction with friends/family, *US* Satisfaction with university

**Table 3:** Discriminant validity of the EPSSEU items

Itens	Score	FFS	US	Total	*p*
Sometimes I feel alone in the world and without	4 (2–5)	**0.581**	0.172	0.553	< 0.001^*^
I feel that I can count on concrete help from the people I live with during the pandemic	4 (3–5)	**0.638**	0.258	0.613	< 0.001^*^
I have several people to talk to, even by phone, messaging apps or social networking, in case I feel lonely	4 (2–5)	**0.594**	0.279	0.560	< 0.001^*^
I am satisfied with the way the people at home are dealing with the pandemic	4 (2–4)	**0.615**	0.264	0.611	< 0.001^*^
My family members have helped me in whatever I need during the pandemic	4 (3–5)	**0.599**	0.284	0.587	< 0.001^*^
My friends have helped me in whatever I need during the pandemic	4 (3–5)	**0.559**	0.294	0.555	< 0.001^*^
I feel that my professors are looking for a way to maintain my learning and bond with the university during the pandemic	4 (3–4)	0.285	**0.936**	0.460	< 0.001^*^
I feel that the university is looking for a safe and efficient way to maintain my learning and bonding during the pandemic	4 (3–4)	0.280	**0.934**	0.463	< 0.001^*^
I have received the financial support I need during the pandemic	4 (3–5)	**0.559**	0.228	0.549	< 0.001^*^

Spearman Correlation (1% significance level)

*Score* Median, *FFS* Satisfaction with friends/family, *US* Satisfaction with university

^*^P value was equal for item correlations with FFS, US, and total

Table 4 presents the correlations between the EPSSEU and ESSS. It was hypothesized that there would be high correlations between the total scores of both scales, as well as between the FFS dimensions of the EPSSEU with the friends and family dimensions of the ESSS. Moderate correlations were expected for the remaining analyses. Although all the correlations were significant, only the correlation between the FFS (EPSSEU) and total score (ESSS) was high.

**Table 4:** A priori hypotheses and results for construct validity using correlation between EPSSEU and ESSS

**Hypotheses**	**Comparison**		***r***	***p***
	**EPSSEU**	**ESSS**		
**High convergent validity expected between similar constructs** **Expected correlation *r* > 0,5**	Total score	Total score	0.4985	< 0.001
	FFS	Friends score	0.3701	< 0.001
	FFS	Family score	0.2739	< 0.001
**Expected moderate convergent validity between items** **Expected correlation *r* > 0,3**	Total score	Friends score	0.3497	< 0.001
	Total score	Family score	0.2533	< 0.001
	FFS	Total score	**0.5366**	< 0.001

Spearman’s correlation (1% significance level)

*EPSSEU* Scale of perceived social support for college students, *ESSS* Satisfaction with Social Support Scale, *FFS* Satisfaction with Friends/Family Support

Table 5 presents the correlation between the scores of the total scale and those of the EPSSEU subscales. It can be seen that the subscale that best explains satisfaction with perceived social support is the one related to friends/family, which accounts for more than two-thirds of the variance of the total scale.

**Table 5:** Correlation between scores of the subscales and total scale

Scale and subscales EPSSEU	FFS	US	*P*
Total scale	0.9563	0.4898	< 0.001^*^
Satisfaction with friends/family		0.2974	< 0.001^*^

Spearman’s Correlation (1% significance level)

*FFS* Satisfaction with friends/family, *US* Satisfaction with university

^*^The *p*-value was equal for the correlations FFS and total scale, and US and total scale

Significant differences were found regarding the presence or absence of anxiety, depression, and stress symptoms in the total scale and subscales of both the EPSSEU (Table 6) and ESSS (Table 7). Individuals who did not have symptoms of anxiety, depression, or stress scored higher, demonstrating that a higher perception of social support is related to the absence of these symptoms, and that the results follow a similar trend between the two scales.

**Table 6:** Known group analysis between the EPSSEU scales and the presence of symptoms of anxiety, depression and stress (DASS-21)

	Anxiety		*p*	Depression		*p*	Stress		*p*
	Yes (183)	No (195)		Yes (179)	No (199)		Yes (172)	No (206)	
Sscore Total	24 (19–27)	28 (24–32)	< 0.001	23 (18–27)	29 (25–32)	< 0.001	24 (19–27)	28 (24–32)	< 0.001
Score FFS	17 (13–20)	21 (17–24)	< 0.001	17 (13–19)	21 (18–24)	< 0.001	16.5 (13–19)	21 (18–24)	< 0.001
Score US	7 (5–8)	8 (6–9)	0.027	7 (5–8)	8 (6–10)	< 0.001	7 (5–8)	8 (6–9)	< 0.001

Mann–Whitney test

*FFS* Satisfaction with friends/family, *US* Satisfaction with university

^*^Data presented as median (p25-p75) for non-parametric variables

**Table 7:** Known group analysis between the ESSS scale and the presence of symptoms of anxiety, depression and stress (DASS-21)

	Anxiety		*p*	Depression		*p*	Stress		*p*
	Yes (124)	No (129		Yes (120)	No (133)		Yes (113)	No (140)	
Total score	41.59 ± 7.85	46.41 ± 8.90	< 0.001^1^	40.57 ± 7.71	47.18 ± 8.43	< 0.001^1^	41.11 ± 7.79	46.41 ± 8.75	< 0.001^1^
Satisfaction with friends score	13.85 ± 1.87	14.71 ± 1.93	< 0.001^1^	13.8 ± 1,90	14.73 ± 1.89	< 0.001^1^	13.74 ± 1.97	14.73 ± 1.83	< 0.001^1^
Satisfaction with family score	10 (7–12)	12 (9–13)	0.0026^2^	10 (6.5–12)	11 (9–13)	0.0014^2^	10 (6–12)	11 (8–13)	0.0017^2^

^*^Data presented as median (p25-p75) and for non-parametric variables and mean ± standard deviation for parametric ones

^1^Student’s t-test

^2^Mann-Whitney test

## Discussion

A stable two-factor solution (dimensions) was identified, corresponding to satisfaction with US and FFS. These dimensions, especially the FFS, were correlated with the scale’s total score and with each other. The scale performed well with regard to reliability and validity. The highest EPSSEU scores, characterized by greater social support, were found in the absence of symptoms of anxiety, depression, and stress.

Other instruments, such as the simplified version of the multidimensional scale of perceived social support, validated in a group of Chinese university students found a 3-factor structure, explaining 77.65% of the variance [42]. In our study, support from friends/family was merged into a single factor, whereas in Guan’s study, the sub-scales are presented separately. While several instruments are used to measure social support [43], there is great diversity in the dimensions assessed, owing to analytical and theoretical differences regarding the constructs [27].

Regarding factor analysis, good construct validity was demonstrated. The KMO index confirmed a pattern of true correlation between the items, and the CFI and TLI indicated a good model fit. However, we cannot ignore that the RMSEA did not reach an acceptable value, but studies indicate that the RMSEA index does show inconsistency, by varying according to the standardized factor loadings, and may indicate an acceptable fit only with a very high measurement quality [44]. Thus, discriminant validity was tested, which showed that the items correlated well with their domain scores. The reliability of the total score was also satisfactory in the internal consistency analysis for the Cronbach’s alpha and McDonald’s ω coefficients.

Furthermore, convergent validity is a good method for assessing the validity by correlating the focal instrument with another instrument that assesses a similar construct [45]. There was a significant correlation, but it was not very strong because the instruments had distinct items,so, the moderate correlation can be explained by the fact that the EPSSEU was adapted to fit the support situation in a context of social restrictions, which was precisely its objective, though it differed substantially in terms of the items assessed.

The COVID-19 pandemic has had a strong impact on the mental health of the population. A study of Chinese university students during COVID-19 showed a 34%, 21%, and 11% prevalence of acute stress, depression, and anxiety symptoms, respectively. In addition, individuals with low perceived social support were 4.84 to 5.98 times more likely than those with high perceived social support to have symptoms of anxiety and depression [46]. Another study on university students in France, confined during the pandemic, sought to assess factors associated with mental health disorders, and found a 11%, 22%, 24%, 16% and 27% prevalence of suicidal thoughts, severe distress, high level of perceived stress, major depression, and high levels of anxiety, respectively. Among the identified risk factors, reporting a mental health outcome was associated with a poor quality of social relationships [47]. Therefore, owing to the relationship between perceived social support and symptoms of anxiety, depression, and stress, the EPSSEU and ESSS scales were compared with the DASS-21 scale, that similarly showed a higher perception of support among individuals without these disorders, indicating the same trend of results between both the scales.

In fact, previous studies have reported that adequate social support has a positive effect on health [48]. A study with university students in Spain showed that higher levels of social support reduced anxiety levels during the COVID-19 pandemic [49]. Another US study on young adults stated that social support from family, in addition to partners or friends, can decrease the severity of mental illness [50]. In general, studies have assessed anxiety and depression as consequences of perceived social support [51], but in our understanding, these conditions may also influence perceived social support, acting in a bidirectional manner.

This study has the limitation of online data collection, but this format was the most feasible possibility in the pandemic and social isolation context. The vast majority of studies during the pandemic were conducted online, using a self-report for convenience. In this sense, a convenience sample from a single university may not effectively represent the university population.

Despite these limitations, the introduction of a robust instrument to assess the perception of social support during periods of social restriction (although, its perception does not necessarily consider social contact), is of considerable importance, given that, it was developed and evaluated with a large sample of the university community. The sample used, either at T0 or T2, meets the minimum requirement for factor analyses, viz., must be greater than or equal to 100, or include at least five times as many observations as the number of variables to be analyzed, with the most acceptable size being a ratio of ten to one (10:1) [35], for providing a reliable validity analysis.

In this study, it was shown that the EPSSEU has evidence of validity in a sample of students from a public Brazilian university during the COVID-19 pandemic, and can be used to assess the perception of support in situations of social restrictions, as experienced in the last two years.

### Supplementary Information

Supplementary Material 1.

## Data Availability

No datasets were generated or analysed during the current study.
