# Supplementary material for: Development and validity evidence on the scale of perceived social support for university students (EPSSEU) during the period of social restrictions
Source: BMC Public Health. 2024 Jun 1;24:1474. doi: 10.1186/s12889-024-18882-3 (PMC11144316; doi:10.1186/s12889-024-18882-3)
Supplement: Supplementary file 1 — Supplementary Material 1. [file 12889_2024_18882_MOESM1_ESM.docx]

**Appendices**

Scale of Perceived Social Support for University Students (EPSSEU)

| According to your opinion or feeling, score your answer on a scale of 1 to 5 points as indicated: | | | | | |
| --- | --- | --- | --- | --- | --- |
|  | **I agree** | | **Don't agree or disagree** | **I disagree** | |
|  | **Totaly** | **For the most part** |  | **For the most part** | **Totaly** |
| Sometimes I feel alone in the world and without support. | 1- ( ) | 2-( ) | 3- ( ) | 4- ( ) | 5- ( ) |
| I feel that I can count on concrete help from the people I live with during the pandemic. | 1- ( ) | 2-( ) | 3- ( ) | 4- ( ) | 5- ( ) |
| I have several people to talk to, even by phone, messaging apps or social networking, in case I feel lonely. | 1- ( ) | 2-( ) | 3- ( ) | 4- ( ) | 5- ( ) |
| I am satisfied with the way the people in my household are dealing with the pandemic. | 1- ( ) | 2-( ) | 3- ( ) | 4- ( ) | 5- ( ) |
| My family members have helped me in whatever I needed during the pandemic. | 1- ( ) | 2-( ) | 3- ( ) | 4- ( ) | 5- ( ) |
| My friends have helped me in whatever I needed during the pandemic. | 1- ( ) | 2-( ) | 3- ( ) | 4- ( ) | 5- ( ) |
| I feel that my professors are looking for a way to maintain my learning and bond with the university during the pandemic. | 1- ( ) | 2-( ) | 3- ( ) | 4- ( ) | 5- ( ) |
| I feel that the university is looking for a safe and efficient way to maintain my learning and bonding during the pandemic. | 1- ( ) | 2-( ) | 3- ( ) | 4- ( ) | 5- ( ) |
| I have received the financial support I need during the pandemic. | 1- ( ) | 2-( ) | 3- ( ) | 4- ( ) | 5- ( ) |
